# Coagulopathy, injury severity and bleeding progression but not prior antiplatelet and anticoagulation therapies drive prognosis in patients with moderate to severe traumatic brain injury

**DOI:** 10.3389/fneur.2025.1592583

**Published:** 2025-07-09

**Authors:** Franziska Lieschke, Daniel T. Marggrander, Konstantin D. Kohlhase, Daniel Touma, Jan Hendrik Schaefer, Sarah C. Reitz, Juergen Konczalla, Cora R. Schindler, Christian Grefkes, Ferdinand O. Bohmann

**Affiliations:** ^1^Department of Neurology, University Hospital, Goethe University Frankfurt, Frankfurt, Germany; ^2^Department of Anaesthesiology, Intensive Care and Pain Therapy, Sana Hospital Offenbach, Offenbach, Germany; ^3^Department of Pediatric Cardiology and Congenital Heart Disease, Deutsches Herzzentrum der Charité, Berlin, Germany; ^4^Faculty of Medicine, Goethe University Frankfurt, Frankfurt, Germany; ^5^Department of Neurosurgery, University Hospital, Goethe University Frankfurt, Frankfurt, Germany; ^6^Department of Traumatology, Hand and Reconstructive Surgery, University Hospital, Goethe University Frankfurt, Frankfurt, Germany

**Keywords:** traumatic brain injury, anticoagulation, (dual) antiplatelet therapy, intracranial hemorrhage, demographic change, falls, elderly

## Abstract

**Introduction:**

Antiplatelet and anticoagulant therapies complicate the management and outcomes of traumatic brain injury (TBI) patients. This study evaluates clinical profiles and short-term outcomes focusing on prior antihemostatic therapy and tranexamic acid (TXA) use.

**Patients and methods:**

We analyzed TBI patients admitted to University Hospital Frankfurt (2018–2021), assessing demographics, injury characteristics, clinical course, and short-term outcomes. The primary endpoint was hemorrhage progression; secondary endpoints included the modified Rankin Scale (mRS) at discharge, mortality and thromboembolic complications. Regression models identified predictors of functional outcome and mortality.

**Results:**

Among 218 patients (median age 70 years, 35% female, median GCS at admission 7), 44% had prior antiplatelet or anticoagulation therapy. These patients were older, had higher pre-injury mRS scores, and more often sustained TBIs from falls. While hemorrhage progression was similar, they had worse mRS scores (*p* = 0.02) and higher mortality (*p* = 0.002). Coagulopathy (OR 1.11, CI 1.07–1.16, *p* < 0.001), injury severity (OR 2.25, CI 1.51–3.41, *p* < 0.001), and bleeding progression (OR 2.23, CI 1.48–3.41, *p* < 0.001) predicted poor functional outcomes. TXA was more often given to younger, severely injured patients but did not impact outcome.

**Conclusion:**

This study underscores the need for tailored therapeutic approaches to improve survival and functional recovery in patients with pre-injury antiplatelet and anticoagulant therapies.

## Introduction

Traumatic brain injury (TBI) constitutes a leading cause of death and disability worldwide, affecting approximately 69 million people worldwide each year (1–4). In recent decades, the epidemiology of TBI has shifted toward older patients (5, 6). Given the increasing age of TBI patients, comorbidities and the number of patients treated with antiplatelet or anticoagulant therapy rise proportionally (5–7). While in the acute setting of TBI, information on preexisting medication may not always be available at admission, previous studies demonstrated a high frequency of pharmacologically impaired platelet function in TBI patients with unknown preexisting medication (8). This frail patient population is particularly at risk for progressive hemorrhage, which occurs in 30 to over 70% of TBI patients (9–13) and predicts unfavorable clinical outcomes (14–16).

Clinically, hemorrhage progression may present with a decline in neurological status, such as increasing confusion, drowsiness, or focal neurological deficits. Management typically involves careful clinical monitoring and serial imaging. Therapeutic approaches in TBI can only address secondary injury mechanisms which also includes secondary hemorrhage (17–19). To this end, measures such as optimizing hemostasis (e.g., reversal therapies), consistent blood pressure management, and surgical approaches are applied. Regarding the optimization of coagulation, the CRASH−3 trial found that the risk of death was reduced in patients treated with tranexamic acid (TXA) which is why its early administration is currently recommended in patients with mild to moderate TBI, even in patients without prior antihemostatic therapy (20). Prompt recognition and intervention are crucial to improve outcomes as secondary hemorrhage significantly increases the risk of morbidity and mortality in the older patients following TBI.

The aim of the present study was to characterize outcomes following TBI in patients admitted to our hospital with a special focus on pre-existing antiplatelet or anticoagulant therapy and the chosen acute treatment approaches.

## Patients and methods

This retrospective study analyzed data from patients with acute TBI, who were admitted to the Center of Neurology and Neurosurgery at Frankfurt University Hospital, Germany between 2018 and 2021. The study was approved by the institutional ethics committee (2022–913). Written informed consent was waived due to the study’s retrospective design.

We included patients with the diagnosis of acute TBI (ICD-10: S06.0 – S06. A) (21), categorizing TBI as acute if admission occurred within 24 h of the trauma.

The following clinical data were captured from medical records: age; sex; injury mechanism; modified Rankin Scale (mRS) prior to TBI; Glasgow Coma Scale (GCS) at admission, after 24 h and at discharge; Richmond Agitation-Sedation Scale (RASS) at admission and at discharge; severity of TBI classified by the GCS score at presentation after resuscitation (grade I “mild”: GCS score 13–15, grade II “moderate”: GCS score 9–12, and finally grade III “severe”: GCS score≤8); surgical interventions such as craniotomy or hemicraniectomy, implantation of an external ventricular drainage (EVD), lumbar drainage (LD) or intracranial pressure monitoring; previous antiplatelet or anticoagulant therapy; laboratory findings; the application of coagulation reversal therapies and/or measures of coagulation and hemostasis optimization such as prothrombin complex concentrate (PCC), fresh frozen plasma (FFP), TXA, vitamin k, Idarucizumab or Andexanet alfa, the application of platelet or erythrocyte concentrates (PC or EC); and finally imaging evidence of intracranial hemorrhage such epidural hematoma (EDH), subdural hematoma (SDH), subarachnoid hemorrhage (SAH) and intraventricular hemorrhage (IVH) and intracerebral hemorrhage (ICH); as well as an open/penetrating TBI and/or midline shift, and finally the restart of antiplatelet or anticoagulant therapies.

The primary endpoint was the rate of secondary hemorrhage progression defined as hemorrhagic lesion expansion measured in the follow-up imaging.

Secondary endpoints included functional outcome at discharge (mRS), mortality and thromboembolic complications (composite endpoint of pulmonary embolism, myocardial infarction, deep vein thrombosis and cerebral ischemic stroke).

Statistical analyses were performed using GraphPad Prism 10 Version 10.3.1 and GNU R 4.4.1 Ordinal and non-normal continuous data is depicted as median and corresponding interquartile range (IQR), normally distributed continuous data is depicted as mean ± standard deviation of the mean (SD) or standard error of the mean (SEM). Frequencies of nominal data are depicted as percentages. Data was assessed for normal distribution using the Kolmogorov–Smirnov-test. Intergroup differences of the included characteristics were evaluated using the *t* test, Mann–Whitney U test, *χ*^2^ or fisher’s test, or using the Kruskal-Wallis test if more than two groups were compared. A *p*-value < 0.05 was set as level of significance. To account for patient age, sex, and injury severity (GCS score at admission, TBI grade and evidence of open/penetrating mechanism), propensity score matching was applied. For predictor analysis, ordered multinomial and logistic regression models were performed.

## Results

### Study population

**Figure 1:**
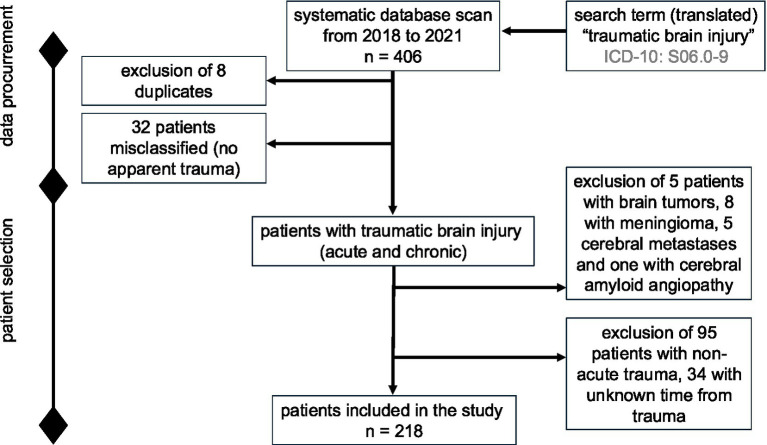
Study flowchart describing the data procurement and stepwise patient selection process intended for retrospective analysis.

The search based on the inclusion criteria yielded 406 patients. After excluding 188 patients (8 duplicates; 5 with brain tumors, 8 with meningeomas, 5 with cerebral metastases, 1 with cerebral amyloid angiopathy, 32 patients without identifiable trauma, 95 with non-acute trauma and 34 with unknown latency between trauma and admission), the final analysis included 218 patients with acute TBI (Figure 1).

The median age of the cohort was 70 (IQR 50–81) years, 76 (34.9%) patients were female. In 119 cases (54.6%), the injury mechanism was unspecified; however, falls accounted for 79 cases (36.2%). The median GCS score at admission was 7. Additional baseline characteristics are detailed in Table 1.

**Table 1:** Patient demographics, injury characteristics and clinical course depending on prior antihemostatic medication status.

Patient characteristics n (%) unless otherwise stated		All (*n* = 218)	No prior anti-hemostatic therapy (*n* = 119)	Prior anti-platelets* (*n* = 54)	Prior anti-coagulants (*n* = 42)	*p*-value
Age (years)		70 (50–81)	55 (39–70)	78 (66–83)	83 (78–85)	**<0.0001**
Sex	Male	142 (65.1%)	83 (69.8%)	37 (68.5%)	20 (47.6%)	**0.03**
	Female	76 (34.9%)	36 (30.2%)	17 (31.5%)	22 (52.4%)	
Mechanism of injury	Fall(s)	79 (36.2%)	27 (22.7%)	26 (48.1%)	23 (54.7%)	**<0.0001**
	Traffic accident	14 (6.4%)	13 (10.9%)	0 (0%)	1 (2.4%)	
	Unknown	119 (54.6%)	75 (63.0%)	28 (51.9%)	16 (38.1%)	
	Secondary TBI**	3 (1.4%)	2 (1.7%)	0 (0%)	1 (2.4%)	
	Physical assault	2 (0.9%)	2 (1.7%)	0 (0%)	0 (0%)	
	Gunshot	1 (0.5%)	0 (0%)	0 (0%)	1 (2.4%)	
GCS	At admission	7 (3–14)	4 (3–11)	9 (3–15)	12 (3–15)	**0.002**
	After 24 h	3 (3–14)	3 (3–12)	6 (3–15)	3 (3–15)	**0.01**
	At discharge	13 (3–15)	13 (3–15)	14 (3–15)	14 (3–15)	0.85
RASS	At admission	-3 (−4 - -1)	-4 (−4 - -1)	−2 (−4–0)	−3 (−4 - − 1)	**0.003**
	At discharge	0 (−3–0)	0 (−3–0)	-1 (−2–0)	0 (−2–0)	0.92
Prior mRS		0 (0–1)	0 (0–0)	1 (0–3)	3 (1–4)	**<0.0001**
TBI grade		3 (1–3)	3 (2–3)	3 (1–3)	2 (1–3)	**0.002**
	1	72 (33%)	28 (23.5%)	24 (45.3%)	20 (48.8%)	**0.006**
	2	13 (6%)	8 (6.7%)	2 (3.8%)	3 (7.3%)	
	3	130 (59.6%)	83 (69.8%)	27 (50.9%)	18 (43.9%)	
Surgical interventions	Surgery***	181 (83%)	100 (84.0%)	44 (81.5%)	35 (83.3%)	0.88
	EVD	22 (10.1%)	12 (10.1%)	8 (14.8%)	2 (4.8%)	0.27
	LD	1 (0.5%)	0 (0%)	0 (0%)	1 (2.4%)	0.2
	ICP monitoring	143 (65.6%)	88 (74%)	30 (55.6%)	24 (57.1%)	**0.02**
Laboratory findings	Platelets (/nl)	198 ± 83	192 ± 72	205 ± 78	212 ± 111	0.84
	aPTT (s)	29 ± 9	27 ± 5	29 ± 5	34 ± 18	**0.0003**
	INR	1.1 (1.02–1.2)	1.1 (1–1.2)	1.1 (1–1.1)	1.3 (1.1–1.9)	**<0.0001**
Imaging characteristics	Open/penetrating	73 (33.5%)	48 (40.3%)	13 (24.1%)	12 (28.6%)	**0.04**
	Midline shift	143 (65.6%)	73 (61.4%)	39 (72.2%)	29 (69.1%)	0.46
	Presence of intracranial hemorrhage	212 (97.3%)	116 (97.5%)	52 (96.3%)	42 (100%)	0.1
Hematoma subtypes	EDH	35 (16.1%)	27 (22.7%)	4 (7.4%)	4 (9.5%)	**0.01**
	SDH	183 (83.9%)	95 (79.8%)	48 (88.9%)	38 (90.5%)	0.22
	ICH	143 (65.6%)	86 (72.3%)	30 (55.6%)	26 (61.9%)	**0.04**
	IVH	70 (32.1%)	39 (32.8%)	18 (33.3%)	13 (31%)	0.96
	SAH	148 (67.9%)	86 (72.3%)	33 (61.1%)	27 (64.3%)	0.38
Reversal therapies	PCC	55 (25.2%)	21 (17.7%)	8 (14.8%)	26 (61.9%)	**<0.0001**
	FFP	10 (4.6%)	8 (6.7%)	0 (0%)	1 (2.4%)	0.1
	PC	31 (14.2%)	23 (19.3%)	6 (11.1%)	2 (4.8%)	**<0.05**
	EC	66 (30.3%)	43 (36.1%)	11 (20.4%)	11 (26.2%)	0.1
	Tranexamic acid	100 (45.9%)	61 (51.3%)	22 (40.7%)	16 (38.1%)	0.23
	Vitamin K	41 (18.8%)	21 (17.7%)	2 (3.7%)	18 (42.9%)	**<0.0001**
	Idarucizumab	0 (0%)	0 (0%)	0 (0%)	0 (0%)	
	Andexanet alfa	0 (0%)	0 (0%)	0 (0%)	0 (0%)	
(Re)start of during stay	Antiplatelets	14 (6.4%)	1 (0.8%)	11 (20.4%)	2 (4.8%)	**<0.0001**
	Anticoagulation	1 (0.5%)	0 (0%)	0 (0%)	1 (2.4%)	0.19
	Thromboprophylaxis	184 (84.4%)	103 (86.6%)	45 (83.3%)	35 (83.3%)	0.78

aPTT, activated partial thromboplastin time; EVD, extraventricular drain; GCS, Glasgow Coma Score; EDH, epidural hematoma; ICH, intracerebral hemorrhage; ICP, intracranial pressure; INR, international normalised ratio; IQR, interquartile range; IVH, intraventricular hemorrhage; LD, lumbar drain; mRS, modified Rankin Scale; RASS, Richmond Agitation-Sedation Scale; SAH, subarachnoid hemorrhage; SDH, subdural hematoma; TBI, traumatic brain injury; TXA, tranexamic acid. *p*-values indicating statistical significance are displayed in bold font.

*Including dual antiplatelet therapy but no patients with additional anticoagulation, these were allocated to the anticoagulant group.

**Secondarily to epileptic seizure or syncope.

***Craniotomy/burr hole.

### Previous antihemostatic therapy

Of the total cohort, 96 patients (44%) had prior antiplatelet or anticoagulant therapy. In detail, 62 patients (28.4%) were on antiplatelet therapy, with 9 (4.1%) receiving dual antiplatelet therapy (DAPT). Among the patients with prior antiplatelet therapy, 8 also received anticoagulant therapy 3 with the vitamin k antagonist (VKA) phenprocoumon (Marcumar ®), 4 with direct oral anticoagulants (DOACs) and 1 with a therapeutic dose of low molecular weight heparin (LMWH). A total of 42 patients (19.3%) received prior anticoagulant therapy (including the aforementioned 8 patients with concomitant antiplatelet therapy), there were 13 patients on VKA, 24 on DOACs and 5 on therapeutic LMWH or unfractionated heparin (UFH). Patients on DOACs, LMWH, UFH and VKA were grouped together as receiving “prior anticoagulant therapy.” Data on previous medication was not available in 3 patients, who were excluded from the subsequent analysis.

### Patient and injury characteristics by antihemostatic therapy status

Patients with prior antiplatelet or anticoagulant therapy were significantly older and had higher pre-injury mRS scores, particularly those on anticoagulant therapy (*p* < 0.0001; Table 1). Falls were more frequently the cause of TBI in these groups (*p* < 0.0001). Sex distribution differed slightly: females were under-represented in patients on antiplatelet therapy or no prior medication, but more prevalent among those on anticoagulation. As expected, these patients showed the highest values in INR and aPTT (*p* < 0.0001 and *p* = 0.0003, respectively). Patients on prior anticoagulant therapy more frequently received PCC and vitamin k (*p* < 0.0001).

Patients without prior antihemostatic therapy exhibited more severe injuries, as reflected by lower GCS scores at admission and after 24 h (*p* = 0.002 and *p* = 0.01, respectively), as well as higher TBI grades (*p* = 0.002). Accordingly, we found significantly more EDH and ICH in patients without prior antihemostatic therapy (*p* = 0.01 and 0.04, respectively) compared to patients on prior anticoagulant or antiplatelet therapy. Most patients across all groups underwent some form of surgical intervention, with significantly more ICP monitoring in patients without prior antihemostatic therapy (*p* = 0.02). Thrombosis prophylaxis with low-molecular-weight heparin (subcutaneous) was also carried out equally frequently during the hospital stay. Antiplatelet therapy was resumed in 20.4% of the patients with prior antiplatelet therapy, whereas anticoagulant therapy was restarted in only one case of the prior anticoagulant group.

### Hemorrhage progression and functional outcomes

**Figure 2:**
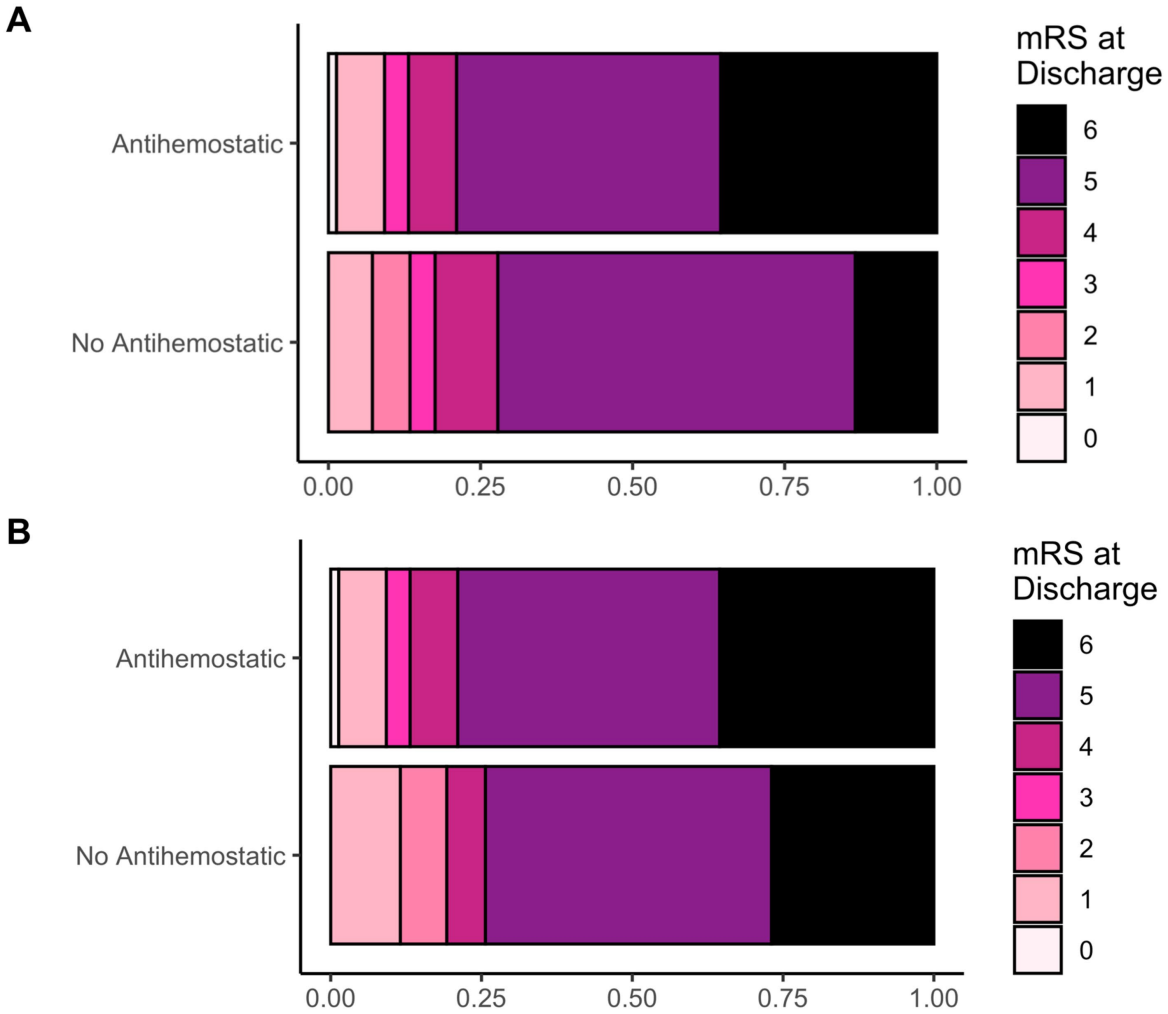
**(A)** Frequency distribution of the neurological outcome based on mRS at discharge (displayed as fractions) in patients with or without prior antihemostatic therapy (antiplatelet and anticoagulant together) and **(B)**; after propensity score matching.

The overall occurrence of hemorrhage progression did not differ significantly by prior antihemostatic therapy. However, we observed worse functional outcomes (higher mRS scores) in patients on prior antiplatelet or anticoagulant therapy (*p* = 0.02; Table 2; Figure 2A). Intrahospital mortality was significantly higher in these patients (*p* = 0.002). Thromboembolic complications were similar across groups.

**Table 2:** Primary and secondary outcomes.

Patient characteristics n (%) unless otherwise stated	All (*n* = 218)	No prior anti-hemostatic therapy (*n* = 119)	Prior anti-platelets* (*n* = 54)	Prior anti-coagulants (*n* = 42)	*p*-value
Hemorrhage progression	86 (39.5%)	41 (34.5%)	24 (44.4%)	21 (50%)	0.15
mRS at discharge	5 (5–6)	5 (4–5)	5 (5–6)	5 (5–6)	**0.02**
Mortality	45 (20.6%)	15 (12.6%)	15 (27.8%)	15 (35.7%)	**0.002**
Thromboembolic complications	26 (11.9%)	11 (9.2%)	7 (13%)	8 (19.1%)	0.23

mRS, modified Rankin Scale. *p*-values indicating statistical significance are displayed in bold font.

*Including dual antiplatelet therapy but no patients with additional anticoagulation, these were allocated to the anticoagulant group.

After propensity score matching for patient age, sex, GCS score at admission, TBI grade and open/penetrating injuries, mRS scores at discharge and mortality were no longer significantly different (Table 3; Figure 2B), while thromboembolic complications now reached statistical significance.

**Table 3:** Primary and secondary outcomes after PSM.

Patient characteristics n (%) unless otherwise stated	No prior anti-hemostatic therapy (*n* = 78)	Prior anti-platelets* (*n* = 42)	Prior anti-coagulants (*n* = 34)	*p*-value
Hemorrhage progression	41 (52.6%)	19 (45.2%)	20 (58.8%)	0.49
mRS at discharge	5 (4–6)	5 (5–6)	5 (5–6)	0.26
Mortality	21 (26.9%)	13 (31%)	14 (41.2%)	0.36
Thromboembolic complications	3 (3.9%)	6 (14.3%)	8 (24.2%)	**0.006**

mRS, modified Rankin Scale. *p*-values indicating statistical significance are displayed in bold font.

*Including dual antiplatelet therapy but no patients with additional anticoagulation, these were allocated to the anticoagulant group.

### Predictors of functional outcomes and mortality

Ordered multinomial regression identified the following predictors of unfavorable outcomes (higher mRS scores): female sex (OR 2.18, CI 1.41–3.41, *p* = 0.001), prolonged aPTT at admission (OR 1.11, CI 1.07–1.16, *p* < 0.001), TBI grade (OR 2.25, CI 1.51–3.41, *p* < 0.001), midline shift (OR 1.63, CI 1.05–2.53, *p* = 0.03), intraventricular hemorrhage (OR 1.84, CI1.22–2.79, *p* = 0.004) and bleeding progression (OR 2.23, CI 1.48–3.41, *p* < 0.001, Table 4).

**Table 4:** Predictors of poor outcome at discharge (ordered multinomial regression model).

Risk factor	Odds ratio (OR)	95%-CI	*p* value
Age	1.01	1.0–1.03	0.05
Female sex	2.18	1.41–3.41	**0.001**
Prior antiplatelets	1.33	0.8–2.21	0.27
Prior anticoagulant	1.29	0.68–2.47	0.44
Platelets	1.0	1.0–1.0	0.28
aPTT	1.11	1.07–1.16	**<0.001**
INR	0.97	0.74–1.44	0.83
GCS admission	1.01	0.94–1.08	0.85
TBI grade	2.25	1.51–3.41	**<0.001**
TXA	1.16	0.77–1.74	0.47
Hemorrhage progression	2.23	1.48–3.41	**<0.001**
Thromboembolism	1.21	0.7–2.13	0.49
Open TBI	0.96	0.64–1.44	0.85
Intraventricular hemorrhage	1.84	1.22–2.79	**0.004**
Midline shift	1.63	1.05–2.53	**0.03**

Intercepts	Odds ratio (OR)	95%-CI	*p* value
0|1	26.71	2.38–299.75	**0.008**
1|2	108.12	10.48–1115.83	**<0.001**
2|3	151.33	14.67–1560.64	**<0.001**
3|4	195.24	18.91–2015.41	**<0.001**
4|5	324.79	31.14–3387.31	**<0.001**
5|6	3111.63	250.20–38698.66	**<0.001**
Observations: 168 R^2^ Nagelkerke 0.654			

aPTT, activated partial thromboplastin time; CI, confidence interval; GCS, Glasgow Coma Score; INR, international normalised ratio; TBI, traumatic brain injury; TXA, tranexamic acid. *p*-values indicating statistical significance are displayed in bold font.

For mortality, logistic regression identified female sex (OR 11.1, CI 3.59–39.65, *p* < 0.001), TBI grade (OR 3.53, CI 1.21–11.5, *p* = 0.026) and prolonged aPTT (OR 1.28, CI 1.15–1.46, *p* < 0.001) as significant predictors (Table 5).

**Table 5:** Predictors of mortality at discharge (logistic regression model).

Risk factor	Odds ratio (OR)	95%-CI	*p* value
Age	1.03	0.99–1.07	0.15
Female sex	11.10	3.59–39.65	**<0.001**
Prior antiplatelets	3.56	0.96–14.09	0.06
Prior anticoagulant	2.17	0.42–11.54	0.36
Platelets	1.00	0.99–1.01	0.86
aPTT	1.28	1.15–1.46	**<0.001**
INR	1.31	0.55–4.63	0.66
GCS admission	0.98	0.80–1.19	0.83
TBI grade	3.53	1.21–11.50	**0.026**
TXA	1.15	0.40–3.34	0.79
Hemorrhage progression	2.01	0.71–5.90	0.193
Thromboembolism	1.01	0.26–3.55	0.990
Open TBI	1.37	0.47–4.08	0.564
Intraventricular hemorrhage	2.26	0.82–6.57	0.122
Midline shift	1.70	0.50–6.26	0.404
Observations: 168 R^2^ Tjur 0.454			

aPTT, activated partial thromboplastin time; CI, confidence interval; GCS, Glasgow Coma Score; INR, international normalised ratio; TBI, traumatic brain injury; TXA, tranexamic acid. *p*-values indicating statistical significance are displayed in bold font.

### Baseline and injury characteristics by patient sex

In order to further investigate the sex-related effects on functional outcomes and mortality observed in the previous analysis, we conducted a secondary subanalysis in which we compared baseline characteristics and injury patterns between female and male patients. Female patients were on average older (*p* < 0.0001), showed a (non-significant) trend toward higher pre-injury mRS scores (*p* = 0.06), and more frequently received prior anticoagulant therapy (*p* = 0.01). Mechanisms of injury did not differ between sexes (*p* = 0.2); however female patients tended to present with a slightly higher incidence of midline shift (*p* = 0.03). Interestingly, surviving female patients demonstrated greater alertness (higher GCS scores and RASS scores) at discharge compared to males (*p* = 0.003 for the GCS score at discharge and *p* = 0.002 for the RASS score at discharge). Further sex-based differences are presented in Supplementary Table S1.

### TXA administration

Finally, the use of TXA was examined. Patients receiving TXA (*n* = 100) were younger (*p* < 0.05) and had more severe injuries, as indicated by lower GCS scores at admission (*p* = 0.002), after 24 h (*p* < 0.0001) and at discharge (*p* = 0.002); lower RAAS scores at admission (*p* < 0.0001) and higher TBI grades (*p* = 0.0002). Sex distribution and prior antiplatelet or anticoagulant use did not differ. However, in terms of outcome parameters, hemorrhage progression, thromboembolic complications, intrahospital mortality and mRS scores at discharge did not differ significantly between the two groups. Data on TXA administration was missing for 1 patient, and 117 patients did not receive TXA (Table 6).

**Table 6:** Patient and injury characteristics according to use of tranexamic acid (TXA).

Patient characteristics n (%) unless otherwise stated		Tranexamic acid (*n* = 100)	No tranexamic acid (*n* = 117)	*p*-value
Age (years)		65 (47–79)	72 (55–82)	**<0.05**
Sex	Male	70 (70%)	71 (60.7%)	0.16
	Female	30 (30%)	46 (39.3%)	
GCS	At admission	4 (3–10)	9 (3–15)	**0.002**
	After 24 h	3 (3–3)	10 (3–15)	**<0.0001**
	At discharge	5 (3–15)	15 (3–15)	**0.002**
RASS	At admission	−4 (−4 - − 2)	-2 (−4–0)	**<0.0001**
TBI grade		3 (2–3)	3 (1–3)	**0.0002**
Prior antiplatelet use		22 (22%)	32 (27.4%)	0.43
Prior anticoagulant use		16 (16%)	26 (22.2%)	0.3
Hemorrhage progression		46 (46%)	40 (34.2%)	0.1
mRS at discharge		5 (5–6)	5 (4–6)	0.09
Mortality		23 (23%)	22 (18.8%)	1
Thromboembolic complications		16 (16%)	10 (8.6%)	0–14

GCS, Glasgow Coma Score; mRS, modified Rankin Scale; RASS, Richmond Agitation-Sedation Scale; TBI, traumatic brain injury. *p*-values indicating statistical significance are displayed in bold font.

## Discussion

Our tertiary trauma center predominantly manages severe TBIs with traumatic intracranial hemorrhages requiring urgent surgical intervention. However, even within this highly selective cohort, clear differences in the clinical profiles of TBI patients with and without prior antihemostatic therapy were observed. Patients with prior antiplatelet or anticoagulant therapy were older and had higher pre-injury disability as indicated by pre-injury mRS scores, reflecting the common overlap between advanced age, comorbidities, and the need for these medications. Falls were the predominant cause of TBI in this group, consistent with previous studies linking anticoagulant use to fall-related injuries among older populations (22, 23). While the rate of hemorrhage progression did not differ between patients with and without antihemostatic therapy, functional outcomes were worse. As such, patients with prior antiplatelet or anticoagulant therapy also exhibited significantly increased mortality rates, aligning with prior literature emphasizing the heightened risk of poor outcomes in patients with TBI under VKA (24, 25), but also DOACs, which may appear to be safer for this particular patient group (26–28).

After propensity score matching, however only thromboembolic complications remained significantly more frequent in patients with prior antiplatelet or anticoagulant therapy, which is not surprising when considering that these therapies are usually discontinued when traumatic intracranial hemorrhage is detected, at least temporarily. A significant body of literature exists, describing the influence of antiplatelets and anticoagulation therapies on the occurrence of traumatic intracranial bleeding (29–31). It is well established that they increase the risk of intracranial hemorrhage and rebleeding but do not necessarily correlate with poorer outcomes in mild and moderate to severe TBI requiring neurosurgical interventions, which our study now confirms (32–35). However, our findings underline the need for tailored clinical management and prevention strategies in this vulnerable population.

Non-modifiable factors like sex and TBI severity (indicated by TBI grade, IVH and midline shift) consistently predicted poor outcomes, while bleeding progression and coagulopathy (increased aPTT) were key modifiable contributors to mortality and morbidity. Thus, aggressive management strategies to mitigate bleeding complications in trauma patients, as the prevention of bleeding progression and managing coagulopathy remain crucial in improving outcomes (36, 37).

Considering the comparison between patient sexes, it is likely that the strong association of female sex with poorer functional outcomes, particularly reduced survival, observed in the predictor analysis may be attributable to confounding factors such as advanced patient age and pre-existing conditions. However, surviving females demonstrated a higher level of alertness at discharge compared to male patients. Previous literature regarding the impact of sex and gender on TBI outcomes is controversial: Some studies suggest that women of childbearing age may have a better prognosis due to hormonal benefits (38), while other studies show no significant differences or suggest that women may fare worse in certain cases, possibly due to hormonal fluctuations or higher prevalence of psychological consequences such as depression and anxiety disorders (39). Further research is needed to better understand gender- and sex-specific differences and to tailor treatment accordingly.

We here also investigated TXA administration and its potential benefits. This is of relevance as the hemostatic and immune systems interact in response to trauma, where plasmin stimulates complement activation. TXA competitively inhibits the conversion of plasminogen to plasmin, which, in turn, inhibits fibrin degeneration. This inhibition of fibrin degeneration may restrict the inflammatory response that can be provoked by fibrin degradation products. Patients receiving TXA presented with more severe injuries and worse baseline GCS scores. We did not detect a difference in hemorrhage progression or functional outcomes in the TXA group. These findings contradict the results of the CRASH-3 trial, which demonstrated that early TXA administration within 3 h of injury reduces mortality in TBI without increasing thromboembolic risks. However, patients with GCS of 3 and those with bilateral unreactive pupils at baseline were excluded. The effect was greatest for patients with mild and moderate head injuries but not apparent in those with severe head injuries (20). Our data showed that TXA was mainly given to severely affected patients, suggesting selection bias in its administration and explaining the lack of a statistically detectable effect. This emphasizes the need for standardized protocols to ensure that TXA benefits are consistently realized across diverse patient populations, as patients with mild and moderate injury who would benefit most from TXA appear to be less likely to receive it.

Our study has important limitations. At first, as a retrospective, single-center study, the findings are inherently limited by the quality and completeness of the recorded data and are subject to selection bias influencing the cohort composition. In our tertiary care trauma center, many of the patients were secondary transfers requiring surgical intervention, which resulted in predominantly moderate to severe injuries. This reduces the generalizability of the findings to broader populations, including those with milder TBIs managed in non-tertiary settings. The study lacked information on critical variables in a significant proportion of patients. For example, the mechanism of injury was not documented in over half of the cases. The study primarily focused on short-term outcomes, such as intrahospital mortality, functional status at discharge, and immediate complications. Long-term follow-up, which is essential to understanding recovery trajectories, reintegration into daily life, and the sustained impact of therapeutic interventions, was not included. This restricts the ability to draw conclusions about the prolonged effects of factors like antihemostatic therapy or its reversal. Furthermore, information on the individual pharmacological response in the antiplatelet group would be of interest as this might also significantly affect patient outcomes and should be addressed in future studies. Lastly, the study grouped patients with different types of anticoagulants and antiplatelet therapies together, despite their varying mechanisms of action and potential risks. As such, phenprocoumon, DOACs, LMWH, and UFH were pooled for statistical analysis. This approach may obscure nuanced differences in how specific agents impact TBI outcomes and limit the applicability of findings to clinical decision-making for particular drugs.

From a clinical perspective, our findings still reinforce the importance of individualized care for TBI patients, particularly those on antiplatelet or anticoagulant therapy. Early and comprehensive coagulation management, including timely reversal of anticoagulation and cautious thromboprophylaxis, remains critical for optimizing outcomes.

## Conclusion

In conclusion, this study underscores the complex interplay of age, anticoagulation, coagulopathy, and injury severity in determining TBI outcomes. It highlights the need for tailored therapeutic approaches and continued investigation into predictive factors and interventions, including TXA, to improve survival and functional recovery in this vulnerable population.

## Data Availability

The original contributions presented in the study are included in the article/Supplementary material, further inquiries can be directed to the corresponding author/s.

## References

[1] Peeters W Majdan M Brazinova A Nieboer D Maas AIR. Changing epidemiological patterns in traumatic brain injury: a longitudinal hospital-based study in Belgium. Neuroepidemiology. (2017) 48:63–70. doi: 10.1159/000471877, PMID: 28448968

[2] Roozenbeek B Maas AIR Menon DK. Changing patterns in the epidemiology of traumatic brain injury. Nat Rev Neurol. (2013) 9:231–6. doi: 10.1038/nrneurol.2013.22, PMID: 23443846

[3] Ramanathan DM McWilliams N Schatz P Hillary FG. Epidemiological shifts in elderly traumatic brain injury: 18-year trends in Pennsylvania. J Neurotrauma. (2012) 29:1371–8. doi: 10.1089/neu.2011.2197, PMID: 22150090

[4] Dewan MC Rattani A Gupta S Baticulon RE Hung YC Punchak M. Estimating the global incidence of traumatic brain injury. J Neurosurg. (2019) 130:1080–97. doi: 10.3171/2017.10.JNS17352, PMID: 29701556

[5] Dams-O’Connor K Juengst SB Bogner J Chiaravalloti ND Corrigan JD Giacino JT. Traumatic brain injury as a chronic disease: insights from the United States traumatic brain injury model systems research program. Lancet Neurol. (2023) 22:517–28. doi: 10.1016/S1474-4422(23)00065-0 37086742

[6] Giner J Mesa Galán L Yus Teruel S Guallar Espallargas MC Pérez López C Isla Guerrero A. Traumatic brain injury in the new millennium: new population and new management. Neurología. (2019) 37:383–89. doi: 10.1016/j.nrleng.2019.03.024, PMID: 35672125

[7] Baggiani M Guglielmi A Citerio G. Acute traumatic brain injury in frail patients: the next pandemic. Curr Opin Crit Care. (2022) 28:166–75. doi: 10.1097/MCC.0000000000000915, PMID: 35081556 PMC8900998

[8] Prinz V Finger T Bayerl S Rosenthal C Wolf S Liman T. High prevalence of pharmacologically induced platelet dysfunction in the acute setting of brain injury. Acta Neurochir. (2016) 158:117–23. doi: 10.1007/s00701-015-2645-8, PMID: 26611691

[9] Narayan RK Maas AIR Servadei F Skolnick BE Tillinger MN Marshall LF. Progression of traumatic intracerebral hemorrhage: a prospective observational study. J Neurotrauma. (2008) 25:629–39. doi: 10.1089/neu.2007.0385, PMID: 18491950

[10] Oertel M Kelly DF McArthur D Boscardin WJ Glenn TC Lee JH. Progressive hemorrhage after head trauma: predictors and consequences of the evolving injury. J Neurosurg. (2002) 96:109–16. doi: 10.3171/jns.2002.96.1.0109, PMID: 11794591

[11] Kurland D Hong C Aarabi B Gerzanich V Simard JM. Hemorrhagic progression of a contusion after traumatic brain injury: a review. J Neurotrauma. (2012) 29:19–31. doi: 10.1089/neu.2011.2122, PMID: 21988198 PMC3253310

[12] Tong W-S Zheng P Xu J-F Guo YJ Zeng JS Yang WJ. Early CT signs of progressive hemorrhagic injury following acute traumatic brain injury. Neuroradiology. (2011) 53:305–9. doi: 10.1007/s00234-010-0659-8, PMID: 20131047

[13] Chang EF Meeker M Holland MC. Acute traumatic intraparenchymal hemorrhage: risk factors for progression in the early post-injury period. Neurosurgery. (2006) 58:647–56. doi: 10.1227/01.NEU.0000197101.68538.E6, PMID: 16575328

[14] Juratli TA Zang B Litz RJ Sitoci K-H Aschenbrenner U Gottschlich B. Early hemorrhagic progression of traumatic brain contusions: frequency, correlation with coagulation disorders, and patient outcome: a prospective study. J Neurotrauma. (2014) 31:1521–7. doi: 10.1089/neu.2013.3241, PMID: 24738836

[15] Carnevale JA Segar DJ Powers AY Shah M Doberstein C Drapcho B. Blossoming contusions: identifying factors contributing to the expansion of traumatic intracerebral hemorrhage. J Neurosurg. (2018) 129:1305–16. doi: 10.3171/2017.7.JNS17988, PMID: 29303442

[16] Yuan F Ding J Chen H Guo Y Wang G Gao WW. Predicting progressive hemorrhagic injury after traumatic brain injury: derivation and validation of a risk score based on admission characteristics. J Neurotrauma. (2012) 29:2137–42. doi: 10.1089/neu.2011.2233, PMID: 22568757 PMC3419842

[17] Jamjoom AAB Rhodes J Andrews PJD Grant SGN. The synapse in traumatic brain injury. Brain. (2021) 144:18–31. doi: 10.1093/brain/awaa321, PMID: 33186462 PMC7880663

[18] Cash A Theus MH. Mechanisms of blood-brain barrier dysfunction in traumatic brain injury. Int J Mol Sci. (2020) 21:3344. doi: 10.3390/ijms21093344, PMID: 32397302 PMC7246537

[19] Corps KN Roth TL McGavern DB. Inflammation and neuroprotection in traumatic brain injury. JAMA Neurol. (2015) 72:355–62. doi: 10.1001/jamaneurol.2014.3558, PMID: 25599342 PMC5001842

[20] The CRASH-3 trial collaborators. Effects of tranexamic acid on death, disability, vascular occlusive events and other morbidities in patients with acute traumatic brain injury (CRASH-3): a randomised, placebo-controlled trial. Lancet. (2019) 394:1713–23. 31623894 10.1016/S0140-6736(19)32233-0 PMC6853170

[21] World Health Organization (WHO) (2019). International statistical classification of diseases and related health problems (ICD), 10th revision

[22] Peeters W van den Brande R Polinder S Brazinova A Steyerberg EW Lingsma HF. Epidemiology of traumatic brain injury in Europe. Acta Neurochir. (2015) 157:1683–96. doi: 10.1007/s00701-015-2512-7, PMID: 26269030 PMC4569652

[23] Maas AIR Menon DK Manley GT Abrams M Åkerlund C Andelic N. Traumatic brain injury: progress and challenges in prevention, clinical care, and research. Lancet Neurol. (2022) 21:1004–60. doi: 10.1016/S1474-4422(22)00309-X, PMID: 36183712 PMC10427240

[24] Della Pepa GM Covino M Menna G Auricchio AM Polli FM Manno A. Are oral anticoagulants a risk factor for mild traumatic brain injury progression? A single-center experience focused on of direct oral anticoagulants and vitamin K antagonists. Acta Neurochir. (2022) 164:97–105. doi: 10.1007/s00701-021-05066-w, PMID: 34850288

[25] Tykocki T Guzek K. Anticoagulation therapy in traumatic brain injury. World Neurosurg. (2016) 89:497–504. doi: 10.1016/j.wneu.2016.01.063, PMID: 26850974

[26] Batey M Hecht J Callahan C Wahl W. Direct oral anticoagulants do not worsen traumatic brain injury after low-level falls in the elderly. Surgery. (2018) 164:814–9. doi: 10.1016/j.surg.2018.05.060, PMID: 30098813

[27] Santing JAL Lee YX van der Naalt J van den Brand CL Jellema K. Mild traumatic brain injury in elderly patients receiving direct Oral anticoagulants: a systematic review and Meta-analysis. J Neurotrauma. (2022) 39:458–72. doi: 10.1089/neu.2021.0435, PMID: 35057639

[28] Cipriano A Pecori A Bionda AE Bardini M Frassi F Leoli F. Intracranial hemorrhage in anticoagulated patients with mild traumatic brain injury: significant differences between direct oral anticoagulants and vitamin K antagonists. Intern Emerg Med. (2018) 13:1077–87. doi: 10.1007/s11739-018-1806-1, PMID: 29520701

[29] Podolsky-Gondim GG Cardoso R Zucoloto Junior EL Grisi L Medeiros M de Souza SN. Traumatic brain injury in the elderly: clinical features, prognostic factors, and outcomes of 133 consecutive surgical patients. Cureus. (2021) 13:e13587. doi: 10.7759/cureus.13587, PMID: 33815990 PMC8009446

[30] Scotti P Séguin C Lo BWY de Guise E Troquet J-M Marcoux J. Antithrombotic agents and traumatic brain injury in the elderly population: hemorrhage patterns and outcomes. J Neurosurg. (2020) 133:486–95. doi: 10.3171/2019.4.JNS19252 31277068

[31] Vedin T Lundager Forberg J Anefjäll E Lehtinen R Faisal M Edelhamre M. Antiplatelet therapy contributes to a higher risk of traumatic intracranial hemorrhage compared to anticoagulation therapy in ground-level falls: a single-center retrospective study. Eur J Trauma Emerg Surg. (2022) 48:4909–17. doi: 10.1007/s00068-022-02016-8, PMID: 35732809 PMC9712377

[32] O’Donohoe RB Lee HQ Tan T Hendel S Hunn M Mathews J. The impact of preinjury antiplatelet and anticoagulant use on elderly patients with moderate or severe traumatic brain injury following traumatic acute subdural hematoma. World Neurosurg. (2022) 166:e521–7. doi: 10.1016/j.wneu.2022.07.042 35843581

[33] Greuter L Ullmann M Mariani L Guzman R Soleman J. Effect of preoperative antiplatelet or anticoagulation therapy on hemorrhagic complications in patients with traumatic brain injury undergoing craniotomy or craniectomy. Neurosurg Focus. (2019) 47:E3. doi: 10.3171/2019.8.FOCUS19546, PMID: 31675713

[34] Ott MM Eriksson E Vanderkolk W Christianson D Davis A Scholten D. Antiplatelet and anticoagulation therapies do not increase mortality in the absence of traumatic brain injury. J Trauma. (2010) 68:560–3. doi: 10.1097/TA.0b013e3181ad6600, PMID: 20065871

[35] Mathieu F Güting H Gravesteijn B Monteiro M Glocker B Kornaropoulos EN. Impact of antithrombotic agents on radiological lesion progression in acute traumatic brain injury: a CENTER-TBI propensity-matched cohort analysis. J Neurotrauma. (2020) 37:2069–80. doi: 10.1089/neu.2019.6911, PMID: 32312149

[36] Rakhit S Nordness MF Lombardo SR Cook M Smith L Patel MB. Management and challenges of severe traumatic brain injury. Semin Respir Crit Care Med. (2021) 42:127–44. doi: 10.1055/s-0040-1716493, PMID: 32916746

[37] Barletta JF Shirah GR Mangram AJ Sucher JF Hostert SA Bruce K. Reversal of pre-injury factor-Xa inhibitors with prothrombin complex concentrates in patients following traumatic brain injury. Clin Neurol Neurosurg. (2023) 235:108040. doi: 10.1016/j.clineuro.2023.108040, PMID: 37944307

[38] Späni CB Braun DJ Van Eldik LJ. Sex-related responses after traumatic brain injury: considerations for preclinical modeling. Front Neuroendocrinol. (2018) 50:52–66. doi: 10.1016/j.yfrne.2018.03.006, PMID: 29753798 PMC6139061

[39] Mollayeva T Mollayeva S Colantonio A. Traumatic brain injury: sex, gender and intersecting vulnerabilities. Nat Rev Neurol. (2018) 14:711–22. doi: 10.1038/s41582-018-0091-y, PMID: 30397256

