# Supplementary material for: Coagulopathy, injury severity and bleeding progression but not prior antiplatelet and anticoagulation therapies drive prognosis in patients with moderate to severe traumatic brain injury
Source: Front Neurol. 2025 Jul 9;16:1592583. doi: 10.3389/fneur.2025.1592583 (PMC12283275; doi:10.3389/fneur.2025.1592583)
Supplement: Supplementary file 1 [file Data_Sheet_1.docx]

Supplemental Table S1

| **patient characteristics**  n (%) unless otherwise stated | | **females**  (n=76) | **males**  (n=142) | **p-value** |
| --- | --- | --- | --- | --- |
| median age (IQR, years) | | 77 (62-85) | 63 (46-78) | **<0.0001** |
| mechanism of injury | fall(s) | 33 (34.4 %) | 46 (32.4 %) | 0.19 |
|  | traffic accident | 6 (7.9 %) | 8 (5.6 %) |  |
|  | unknown | 34 (44.7 %) | 85 (59.9 %) |  |
|  | secondary TBI* | 2 (2.6 %) | 1 (0.7 %) |  |
|  | physical assault | 1 (1.3 %) | 1 (0.7 %) |  |
|  | gunshot | 0 (0%) | 1 (0.7 %) |  |
| median GCS (IQR) | at admission | 8 (3-15) | 6 (3-13) | 0.2 |
|  | after 24 hours | 3 (3-15) | 3 (3-13) | 0.2 |
|  | at discharge | 15 (7-15) | 9 (3-15) | **0.003** |
| median RASS (IQR) | at admission | -4 (-4 to -1) | -3 (-4 to -1) | 0.5 |
|  | at discharge | 0 (-1 to 0) | -1 (-3 to 0) | **0.002** |
| median prior mRS (IQR) | | 0 (0-3) | 0 (0-1) | 0.06 |
| median TBI grade (IQR) | | 3 (1-3) | 3 (1-3) | 0.2 |
| TBI grade | 1 | 31 (40.8 %) | 41 (28.9 %) | 0.2 |
|  | 2 | 3 (3.9 %) | 10 (7.0 %) |  |
|  | 3 | 42 (55.3 %) | 88 (62 %) |  |
| surgical interventions | surgery** | 64 (84.2 %) | 117 (82.4 %) | 1 |
|  | EVD | 3 (3.9 %) | 19 (13.4 %) | **0.03** |
|  | LD | 1 (1.3 %) | 0 (0 %) | 0.4 |
|  | ICP monitoring | 44 (57.9 %) | 99 (69.7 %) | 0.07 |
| laboratory findings | platelets (mean±SD, /nl) | 203±102 | 187±68 | **0.03** |
|  | aPTT (mean±SD, s) | 27.6±5.4 | 29.8±10.7 | 0.06 |
|  | median INR (IQR) | 1.1 (1.0-1.2) | 1.1 (1.0-1.2) | 0.5 |
| imaging characteristics | open/penetrating | 19 (25 %) | 54 (38.0 %) | 0.07 |
|  | midline shift | 56 (73.7 %) | 87 (61.3 %) | **0.03** |
|  | presence of intracranial hemorrhage | 73 (96.1 %) | 139 (97.9 %) | 1 |
| hematoma subtypes | EDH | 11 (14.5 %) | 24 (16.9 %) | 0.7 |
|  | SDH | 66 (86.8 %) | 117 (82.4 %) | 0.4 |
|  | ICH | 45 (59.2 %) | 98 (69.0 %) | 0.2 |
|  | IVH | 20 (26.3 %) | 50 (35.2 %) | 0.2 |
|  | SAH | 44 (57.9 %) | 104 (73.2 %) | **0.03** |
| prior | antiplatelets*** | 17 (22.4 %) | 37 (26.1 %) | 0.6 |
|  | anticoagulants | 22 (28.9 %) | 20 (14.1 %) | **0.01** |

**Supplementary Table S1:** Patient demographics, injury characteristics and clinical course depending on patient sex. Abbreviations: aPTT= activated partial thromboplastin time, EVD = extraventricular drain, GCS = Glasgow Coma Score, EDH = epidural hematoma, ICH= intracerebral hemorrhage, ICP = intracranial pressure, INR = international normalised ratio, IQR= interquartile range, IVH= intraventricular hemorrhage, LD = lumbar drain, mRS = modified Rankin Scale, RASS = Richmond Agitation-Sedation Scale, SAH= subarachnoid hemorrhage, SDH = subdural hematoma, TBI = traumatic brain injury, TXA = tranexamic acid. P-values indicating statistical significance are displayed in bold font.

* Secondarily to epileptic seizure or syncope

**Craniotomy/burr hole

****Including dual antiplatelet therapy but no patients with additional anticoagulation, these were allocated to the anticoagulant group
